# Synthetic Evaluation of Standard and Microwave-Assisted Solid Phase Peptide Synthesis of a Long Chimeric Peptide Derived from Four *Plasmodium falciparum* Proteins

**DOI:** 10.3390/molecules23112877

**Published:** 2018-11-05

**Authors:** Yahson F. Varela, Magnolia Vanegas Murcia, Manuel Elkin Patarroyo

**Affiliations:** 1 Department of Chemical Synthesis, Fundación Instituto de Inmunología de Colombia (FIDIC), Carrera 50 No. 26-20, Bogotá 111321, Colombia; yfvarelaq@gmail.com; 2 Department of Chemistry, Faculty of Sciences, Universidad Nacional de Colombia, Facultad de Ciencias, Carrera 45 No 26-85, Bogotá 11001, Colombia; 3 School of Medicine and Health Science, Universidad del Rosario, Carrera 24 No 63c-69, Bogotá 111221, Colombia; 4 Director General, Fundación Instituto de Inmunología de Colombia (FIDIC), Carrera 50 No. 26-20, Bogotá 111321, Colombia; mepatarr@gmail.com

**Keywords:** solid phase peptide synthesis, chimeric peptide, coupling, microwave radiation, Fmoc protocol

## Abstract

An 82-residue-long chimeric peptide was synthesised by solid phase peptide synthesis (SPPS), following the Fmoc protocol. Microwave (MW) radiation-assisted synthesis was compared to standard synthesis using low loading (0.20 mmol/g) of polyethylene glycol (PEG) resin. Similar synthetic difficulties were found when the chimeric peptide was obtained via these two reaction conditions, indicating that such difficulties were inherent to the sequence and could not be resolved using MW; by contrast, the number of coupling cycles and total reaction time became reduced whilst crude yield and percentage recovery after purification were higher for MW radiation-assisted synthesis.

## 1. Introduction

Bruce Merrifield developed solid phase peptide synthesis (SPPS) in 1963; this involves a solid support where the peptide becomes elongated and excess reagents and by-products being withdrawn through simple filtration after every reaction step (incorporating α-amino and deprotection) [1,2]. Significant advances have been made in SPPS; however, this methodology has involved difficulties regarding synthesis in some cases, these being very important concerning difficult peptide sequences having severe inefficiencies regarding acylation and deprotection reactions depending on peptide sequence of amino acid (aa) residues. Peptide sequences containing much hydrophobic aa, bulky side chains or bulky protecting groups are more prone to such inefficiencies, thereby producing peptide chain deletion or truncation, in turn, is the product of peptide-resin and/or secondary structure (mostly *β*-sheet) aggregation causing steric hindrance [3]. These associations produce low peptide-resin complex solvation and low reagent accessibility to reaction sites. Such complications could be significant regarding long peptide synthesis because there is the high likelihood of interactions between adjacent chains and steric hindrance; moreover, long peptides require more reaction steps regarding coupling and deprotection reactions, thereby increasing the probability of incomplete reactions [4,5,6].

Polystyrene-polyethylene glycol (PS-PEG), polyethylene glycol-acrylamide (PEGA) [7] and polyethylene glycol (PEG) [8] resins have been used to overcome such synthesis aggregation problems, usually involving great swelling and high solvation directing reagent diffusion toward reaction sites; also, low loading resins have been used to reduce the probability of association. 

Chaotropic agents and pseudo-prolines have been used to avoid these associations and conventional heat and microwave (MW) radiation have been used for disrupting these associations and accelerating synthesis [6,9]. However, such strategies do not ensure such difficulties being overcome because target molecule synthesis also depends on its aa composition which could involve significant steric hindrance and intra-molecular interactions which would lead to inefficient synthesis. 

SPPS protocols highly standardised let produce peptides of high purity, and, average peptide of 50 residues of long, but, also well know that shorter peptide could be very problematic because the sequence composition and longer peptide could be synthesized by this stepwise synthesis like Ribonuclease A was made first time in 1971 [9,10,11]. Two main techniques were introduced to try overcoming the cumulative effect of stepwise synthesis of long peptide sequences and small proteins; protected peptide fragments and chemical ligation. Condensation of the protected peptide was the method commonly used in the 80’s, and can be performed in solution or solid phase; however, this approach provides limited utility because of the racemisation, low solubility and longer reactions times of the protected peptides [12,13]. Chemical ligation (Native chemical ligation, peptide hydrazides, click reaction, a-Ketoacid-hydroxylamine ligation and Staudinger ligation) is a reaction chemoselective, which do not have racemisation problems and better solubility than the approach of the protected peptide; however, both require the purification steps of intermediaries and the final product [14]. On the other hand, stepwise synthesis has let produce these macromolecules by standard and MW-assisted SPPS, and these only require the purification of the final product which simplifies the process; but, MW radiation not only accelerate coupling and deprotection reactions, moreover, could accelerate byproducts formation if the reaction conditions are not controlled [15,16,17,18].

This article reports the synthesis of an 82-residue-long linear peptide chimera (^82^KNSFSLGENPNANPGGVIKHMRFHADYQAPFLGGGYGGEVLYHVPLAGVYRSLKKQLEGGMTDVNRYRYSNNYEEQPHISGG^1^, derived from four protein fragments which are relevant to *Plasmodium falciparum* invasion of target cells [19]) and its evaluation regarding two reaction conditions (standard and MW-assisted) involving a low loading (0.20 mmol/g) PEG resin (Rink Amide ChemMatrix). 

## 2. Results

Loading resin was initially reduced from 0.60 mmol/g to 0.20 mmol/g to minimise the probability of synthesis difficulties caused by aggregation between adjacent chains (*β*-sheet secondary structure) during peptide elongation over the solid support. 

Cleavage controls were made every 11 residues coupled, approximately, to evaluate synthesis progress. A single signal for the 11th residue was observed in the chromatogram during the first standard synthesis control (i.e., its ion peak at 1228.53 Da); this was also observed in the MW-assisted strategy (Table 1, Figure S1). A single signal for the 22nd residue was observed in the chromatogram for the second control of both synthesis conditions (i.e., molecular ion peak at 2628.16 Da; Table 1, Figure S2). Synthesis inefficacy regarding peptide elongation thus did not occur up to this point; SPPS peptides of this length are usually obtained without difficulties because such length is not long enough for adjacent interaction between peptide chains [19].

Three signals appeared in the chromatogram for the third control of the 33rd residue regarding crude peptide synthesised by both reaction conditions (Table 1, Figure S3). Fractions from every peak were collected and analysed by MALDI-TOF MS in each case. Minor peak t_R_ (19.72 min. standard and 19.85 min. MW-assisted) for both reaction conditions was likely due to Gly deletion (residue 23 or 24). The t_R_ of 21.18 min. standard and 21.29 min. MW-assisted were for the 33-residue-long elongated peptide (3887.86 Da). The higher t_R_ (22.43 and 22.26 min. standard and MW-assisted, respectively) ter-butylated cleavage-derived species caused to ter-butyl group junction with the peptide chain; this group came from the aa chain previously protected by t-Boc group. This frequently occurs regarding peptide synthesis cleavage and could be overcome by improving cleavage conditions (time, volume and/or scavenger) [20]. It is worth stressing that the highest intensity peak for both reaction conditions occurred for the 33-residue-long target molecule. 

Three signals were observed in the chromatograms for the fourth control of the 44th residue regarding both reaction conditions; they were for deletion species, the 44-residue-long target molecule and the ter-butylated species (Table 1, Figure S4). The intensity of the peak containing the target molecule was similar to that for deletion species; such decrease in peak intensity (compared to 33-residue-long control) was produced by the accumulation of incompleted acylations and deprotections caused by aggregation, thereby hampering synthesis (observed in both strategies). Similar peak intensity results were obtained for the control for the 57th residue (Figure S5). Decreased intensity regarding control for the 66th residue for both standard and MW-assisted synthesis was observed; two signals appeared in the chromatograms for the 66-residue-long target molecule. During the synthesis, inefficiencies accumulations caused a decrease in intensity for the peak that contained the target molecule (Figure S6).

**Figure 1:**
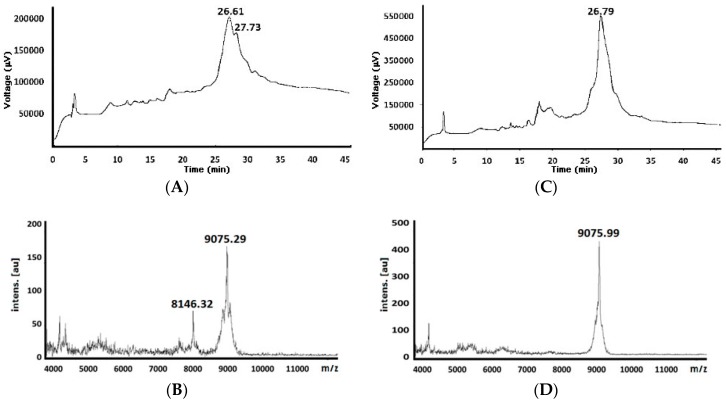
Chromatographic profiles and mass spectra for crude peptide’s 82nd residue (theoretical 9051.42 Da) obtained by standard (**A**) and (**B**) (Chromatogram and mass spectra, respectively) and MW-assisted reaction conditions (**C**) and (**D**) Chromatogram and mass spectra, respectively).

The 82-residue-long chimeric peptide was obtained (^82^KNSFSLGENPNANPGGVIKHMRFHADYQAPFLGGGYGGEVLYHVPLAGVYRSLKKQLEGGMTDVNRYRYSNNYEEQPHISGG^1^) by both strategies; the broad signal was observed in the chromatograms which the target peptide (Figure 1); these chromatograms did not have significant differences regarding both reaction conditions. Total synthesis reaction time was 9720 min (162 h) for the standard strategy compared to 2268 min (37.8 h) for the MW-assisted strategy, giving a 4.26:1 ratio. Shorter MW-assisted reaction time was due to less time being required for every coupling and deprotection reaction step enabling this macromolecule to be obtained fourfold faster than in standard conditions. 

Greater crude peptide yield was obtained by MW-assisted strategy (13.06% compared to 9.98% for the standard condition, Table 2). 

**Figure 2:**
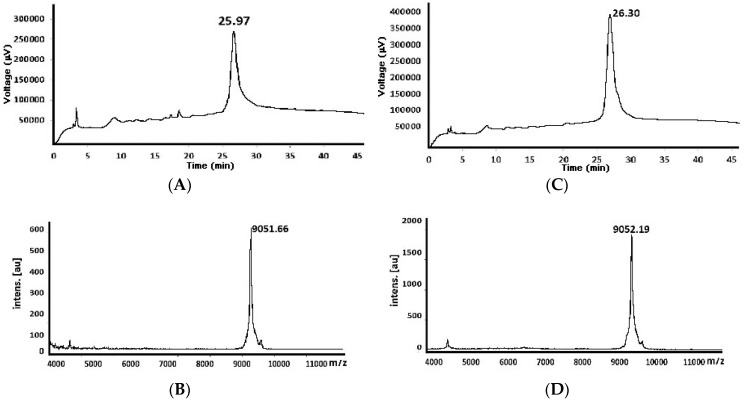
Chromatographic profiles and mass spectrum of the purified peptide’s 82nd residue (MW theoretical 9051.42 Da) obtained by standard (**A**) and (**B**) (Chromatogram and mass spectra, respectively) and MV-assisted reaction conditions (**C**) and (**D**) Chromatogram and mass spectra, respectively).

The 82-residue-long chimeric peptide synthesised by these two strategies was purified (Figure 2); the MW-assisted SPPS had a higher recovery percentage (12.67%). All this demonstrated that although the MW did not totally avoid inefficient synthesis; it was good enough to improve chimeric peptide yield and purity.

In a previous study, the chimera obtained here by MW-assisted synthesis was evaluated in murine BALB/C mice against malaria (*Plasmodium berghei* ANKA and *yoelii* 17XL) and this shows protection induce capacity [21].

## 3. Discussion 

The obtained chimera peptide or 82-mer is remarkable, and how is shown in the results the standard strategy also lets obtained the molecule, but MW-assisted approach produces a better product in yield with less time. Here is important highlight that the stepwise synthesis is a tool important to obtain long peptide and with the advances obtained every day, this technique is gain more field of development. In another side, chemical ligation let also obtained these types of molecules, but require not only personal prepared but also some special equipment. Besides, chemical ligation has some disadvantages (Table 3) as longer times the reaction is required, and, not always let’s obtain peptide bond. Native Chemical Ligation requires a cysteine or alanine residue in a strategic position to obtain the long peptide or protein, also, all techniques of ligation require an additional step of separation between reagents and product. These are the reasons of what is essential follow investigating in stepwise to obtain this long peptide, and in the literature, some landmark peptides like ribonuclease A (124 residues) and the peptide of human immunodeficiency virus (HIV)-1 (Tat of 86 residues) are good are examples of the potential of SPPS [22,23,24].

## 4. Materials and Methods 

### 4.1. Reagents and Solvents

The following reagents, 9-fluorenylmethoxycarbonyl (Fmoc) protected aa, O-(Benzotriazol-1-yl)-*N*,*N*,*N*′,*N*′-tetramethyluronium tetrafluoroborate (TBTU), *N*,*N*′-dicyclohexylcarbodiimide (DDC) and 1-hydroxybenzotrazole (HOBt), were purchased from AAPPTEC (Louisville, KY, USA), ChemMatrix resin (0.60 mmol/g) from PCAS BiomMatrix Inc. (Saint-Jean-sur-Richelieu, QC, Canada), *N*,*N*-diisopropylethylamine (DIPEA), triisopropylsilane (TIS), Triton X-100, α-cyano-4-hydroxycinnamic acid (HCCA), methanol and absolute ethanol from Sigma-Aldrich (St. Louis, MO, USA), piperidine, pyridine, phenol, potassium cyanide, isopropyl alcohol (IPA), acetonitrile (ACN), *N*-methyl-2-pyrrolidone (NMP), trifluoroacetic acid (TFA) and diethyl ether from Panreac (Barcelona, Spain), acetic anhydride (Ac_2_O), ninhydrin and ethanedithiol (EDT) from Merck (Boston, MA, USA) and dichloromethane (DCM) from The Dow Chemical Company (Bogotá, Colombia).

### 4.2. Peptide Design

**Scheme 1:**
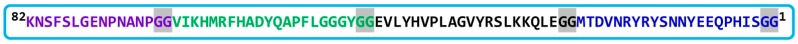
Linear chimeric peptide sequence derived from four *Plasmodium falciparum* proteins (CSP, STARP, MSP-1, and RESA-155; residues marked with letters purple, green, black and blue colour, respectively).

A linear chimeric peptide consisting of four *Plasmodium falciparum* epitopes separated by Gly-Gly (GG) spacers was selected for studying this long peptide’s synthesis pattern (Scheme 1). The glycine is commonly used as a spacer due to its flexibility, low hindrance [25] and experimentally we have found structural similarity between monomer and polymerised peptides (without G and with G spacer, respectively) platforms. The epitopes were selected bearing previous research in mind [19,26,27,28,29], and the Peptide Companion software (free software of excel version, Spyder Institute’s, Praha, Czech Republic) was used for choosing the order of the chimeric peptide’s epitope [30].

### 4.3. Peptide Synthesis 

The chimeric peptide was synthesised by SPPS using Fmoc/tBu protocol on 0.60 mmol/g Rink Amide ChemMatrix resin (PCAS BiomMatrix Inc., Saint-Jean-sur-Richelieu, QC, Canada), using standard and MW-assisted strategies, briefly described below.

Initially, 1.008 g resin (loading: 0.60 mmol/g) was swollen with 5 mL DCM and NMP for 10 min in each solvent; 0.20 mmol Fmoc-Gly-OH was then added to the resin and reacted for 60 min at room temperature (RT); the aa had been previously activated with TBTU/HOBt/DIPEA (1:1:2 mmol) and dissolved in 5 mL NMP. The resin was rinsed with DMF, IPA, and DCM three times with each solvent for 1 min during this time; the amine group in the resin which did not react was then capped with 10 mL acetylation solution (anhydride acetic/DMF/pyridine; 1:1:1) for 60 min at RT and the resin was rinsed again. A qualitative Kaiser’s test was used at this point for evaluating whether an amine group was present [31]; 0.20 mmol/g resin loading was thus obtained, as described by Boll et al. [32]. Fmoc-Gly coupled resin was used in both aforementioned strategies. 

#### 4.3.1. The Standard Strategy 

Fmoc-Gly-resin (loading: 0.20 mmol/g) was deprotected with three cycles of 10 mL piperidine/DMF (1:1) for 10 min. and washed with DMF, IPA and DCM for 1 min, three times. The first strategy for all couplings involved using a fivefold excess of Fmoc aa derivative, DDC/HOBT in NMP. The coupling reaction was carried out for 60 min at RT. A second cycle was carried if the Kaiser test indicated incomplete coupling, using a fivefold excess with TBTU/HOBT/DIPEA. The Fmoc group was removed after every aa had been anchored, as previously described here (acylation and deprotection are evaluated by qualitative Kaiser in every reaction step). Peptides were cleaved with 10 mL/g TFA/TIS/H_2_O/EDT resin (94:1:2.5:2.5) for 3 h at RT; the peptides were then precipitated from the cleavage solution using cold diethyl ether and washed four times with cold ether [33].

#### 4.3.2. The Microwave-Assisted Strategy 

All microwave (MW)-assisted strategy couplings and deprotections were performed at 80 °C for 30 s of radiation (10 Watts) and then for 1 min at RT, except for Asp and His aa coupling and Asp aa deprotection which were carried out at RT for 60 min, as described for the standard strategy, to avoid by-product formation (racemisation and aspartimide) [34]. Cleavage involved the same conditions as for standard synthesis.

### 4.4. Characterization 

#### 4.4.1. HPLC Analysis 

The crude chimeric peptide and reaction control intermediates were analysed by reversed-phase HPLC using a Merck-Hitachi chromatograph with a Vydac C-18 column (250 mm length, 4.6 mm inner diameter, 5 μm particle size, Merck, Kenilworth, NJ, USA). The samples were dissolved in water and acetonitrile (1:1) with 0.05% TFA and eluted with a 0% to 70% gradient of solution B (0.05% TFA in water [Solution A] and 0.05% TFA in acetonitrile [Solution B]) for 45 min at RT and 1 mL/min. Peak signals were detected at 210 nm. 

#### 4.4.2. Mass Spectrometry

A Microflex MALDI-TOF spectrometer (Bruker Daltonics, Billerica, MA, USA) was used for characterising the products; 18.0 μL of an oversaturated α-cyano-4-hydroxycinnamic acid matrix solution was mixed with 2.0 μL peptide solution (5.0 × 10^−4^ mg/μL), and then 2.5 μL of this mixture was placed in the 96-well plates to be analysed.

#### 4.4.3. Purification of the Chimeric Peptide

The 82-mere peptide obtained by the two strategies were purified on a semi-preparative chromatograph (Merck-Hitachi) using a Vydac C-18 Colum (250 mm length, 22 mm inner diameter, 10 μm particle size). Each peptide (1 mL that contained 10 mg approx.) was injected and eluted with 21–41% gradient of solution B for 60 min at RT and 4.5 mL/min of flow. The peak signals were detected at 210 nm; fractions of these were collected, analysed by MALDI-TOF mass spectrometry and to those that contained the target molecule were chosen to analysed by analysis RP-HPLC (Section 4.4.1). Finally, the fractions selected were joined and lyophilised to obtain the purified peptide (Figure 2).

## 5. Conclusions

The present study has described the synthesis of a chimeric peptide by SPPS methodology involving standard and MW-assisted strategies for Fmoc/tBu protocol. Both procedures involved some difficulties caused by the sequence itself thereby hampering synthesis of this 82-residue-long molecule. Although the MW-assisted strategy did not totally overcome such difficulties, it had a shorter reaction time, higher crude product yield and higher final product recovery percentage during purification.

## Figures and Tables

**Table 1:** Results of controls and chimeric peptide for standard and microwave (MW)-assisted synthesis.

Residue Control	Molecular Weight (Da) ^§^	Standard		Microwave-Assisted	
		(*m/z*) ^a^	t_R_ (min)	(*m/z*) ^a^	t_R_ (min)
**11**	1228.53	**1229.52**	14.20	**1230.01**	14.43
**22**	2628.16	**2628.29**	17.17	**2628.06**	17.59
**33**	3887.86	3831.50 (−57.81) ^b^ 3887.90	19.72	3831.93 (−57.52) ^b^ 3889.61	19.85
		**3889.31**	21.18	**3889.45**	21.29
		3888.07 4055.84 (+166.53) ^c^	22.43	3889.18 4058.04 (+168.59) ^c^	22.26
**44**	5065.51	4939.21 (−128. 02) 4996.03 (−71.20) 5066.91	27.36	4938.52 (−128.34) 4995.93 (−70.93) 5067.40	27.50
		**5067.23**	28.60	**5066.86**	28.64
		5065.90 5178.52 (+111.29) ^c^	29.87	5066.98 5178.61 (+111.75) ^c^	29.42
**57**	6348.07	6221.41 (−127.59) 6234.78 (−114.54) 6350.06	28.17	6235.14 (−114.91) 6351.36	27.97
		6291.44 (−57.88) **6349.32**	29.05	**6350.05**	28.66
		6349.47 6632.30 (+282.98)	29.41	ND	29.16
**66**	7467.68	ND	28.65	ND	28.73
		**7469.35** 7580.21 (+111.86) ^c^	29.51	**7467.84**	29.54
**82 ^e^**	9051.42	8146.32 (−928.97) **9075.29** (+23.87) ^d^	26.61 27.73	**9075.99** (+24.57) ^d^	26.79

**^§^** Molecular weight calculated (Table S1). ^a^ The values in bold letters marked for *m/z* of standard and MW-assisted synthesis correspond to molecular ion of the target molecule in the main peak of chromatogram (control residues 11th and 22nd) or the peak of fraction recollected. ^b^ ion peak corresponds to a glycine deletion. ^c^ Values calculated for multiple ter-butylations (t-Bu = 56.1 Da, 2t-Bu = 112.2 Da, 3t-Bu = 168.3Da; Table S2). ^d^ Value calculated for peak ion plus sodium. ^e^ Here fractions did not recollect. Note: the values in parentheses correspond to differences with the expected molecular weight.

**Table 2:** Control synthesis of standard and MW-assisted strategies.

Strategy	Resin (g)	Obtained (mg)		% Yield	Purified Quantity (mg)	Amount of Peptide Obtained in the Purification (mg)	% Purification Yield
		Theoretical	Experimental				
Standard	0.507	905.70	90.42	9.98	10.58	0.97	9.16
MW-assisted	0.501	905.80	118.32	13.06	11.13	1.41	12.67

**Table 3:** Advantages and disadvantages of chemical ligation.

Advantages	Disadvantages	Ref.
Good yield Chemo-selective or high specificity reaction Side-chain protection groups unnecessary NCL produce peptide bond The absence of racemisation at the site ligation	In some case, the reaction typically takes 1–3 days to go to completion The additional purification step is necessary Produce peptide with no natural bond NCL requires cysteine for ligation	[22]

## Supplementary Materials

The following are available online.
