# Supplementary material for: Synthetic Evaluation of Standard and Microwave-Assisted Solid Phase Peptide Synthesis of a Long Chimeric Peptide Derived from Four Plasmodium falciparum Proteins
Source: Molecules. 2018 Nov 5;23(11):2877. doi: 10.3390/molecules23112877 (PMC6278645; doi:10.3390/molecules23112877)
Supplement: Supplementary file 1 [file molecules-23-02877-s001.pdf]

Supplementary Materials: Synthetic Evaluation of Standard and Microwave-Assisted Solid Phase Peptide Synthesis of a Long Chimeric Peptide Derived from Four Plasmodium falciparum Proteins

Yahson F. Varela, Magnolia Vanegas Murcia and Manuel Elkin Patarroyo.

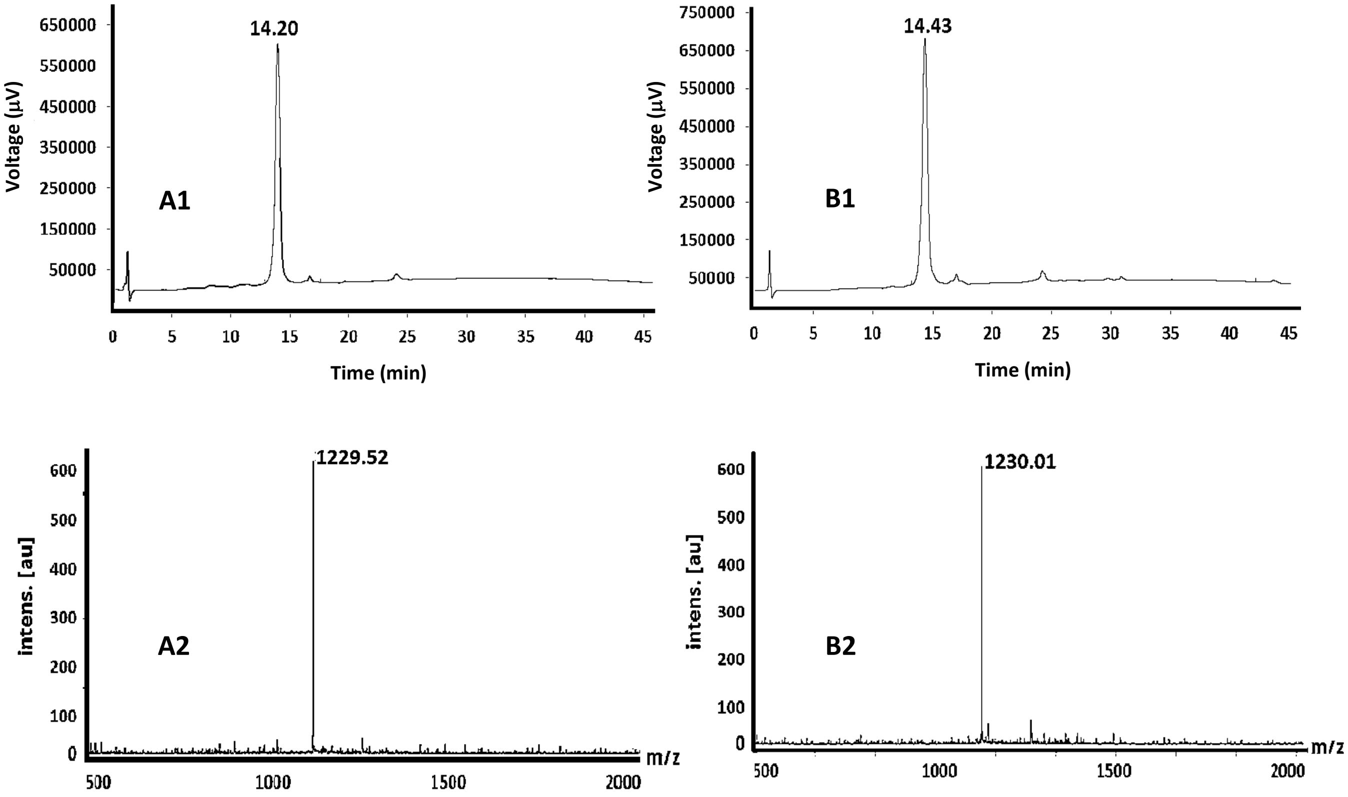

A) B)

Figure S1. Chromatographic profiles of crude peptide's 11th residue and mass spectrum (1,228 Da theoretical) from standard (panel A1 and A2) and microwave-assisted reaction conditions (panel B1 and B2).

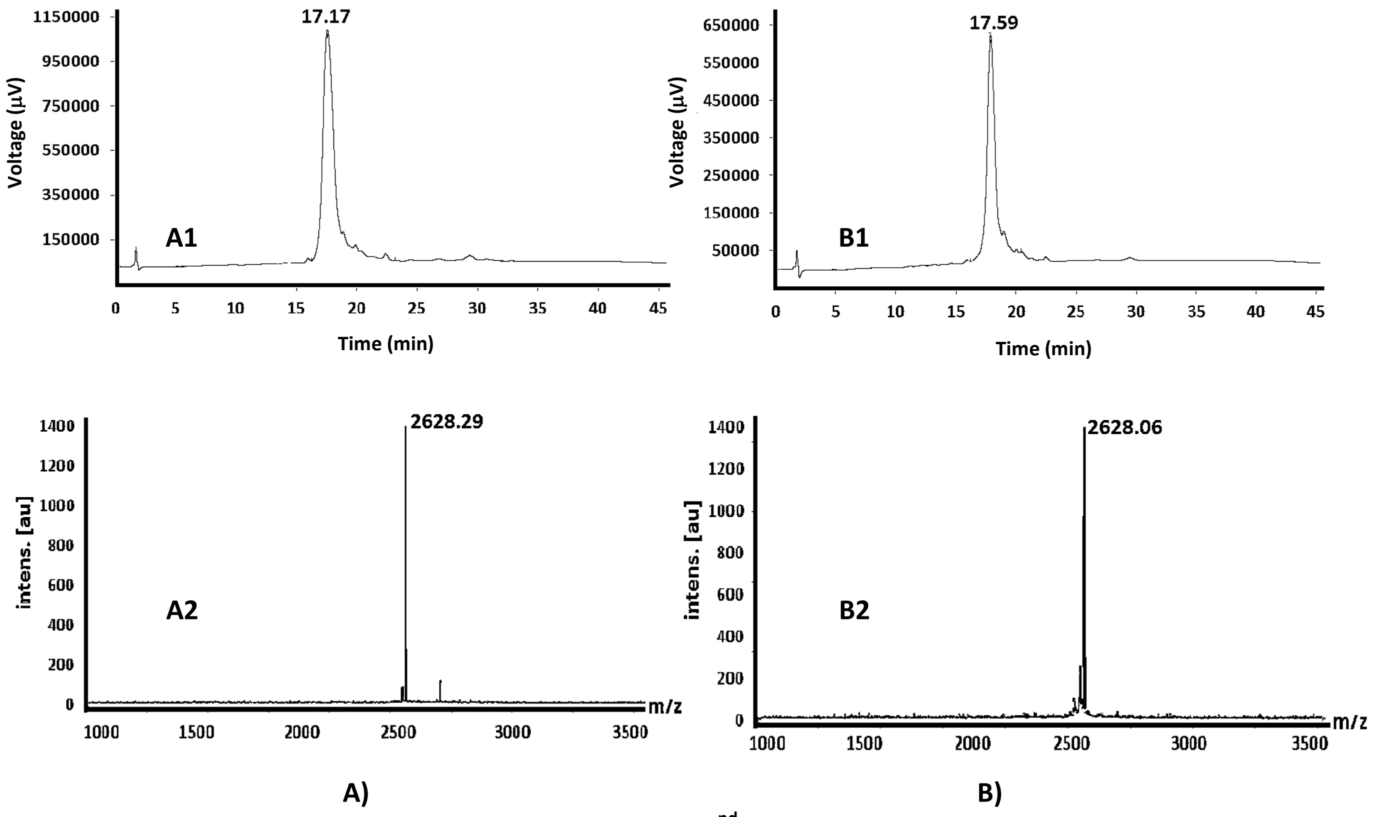

Figure S2. Chromatographic profiles of crude peptide's 22nd residue and mass spectrum (2,628.16 Da theoretical) from standard(panel A1 and A2) and microwave-assisted reaction conditions (panel B1 and B2).

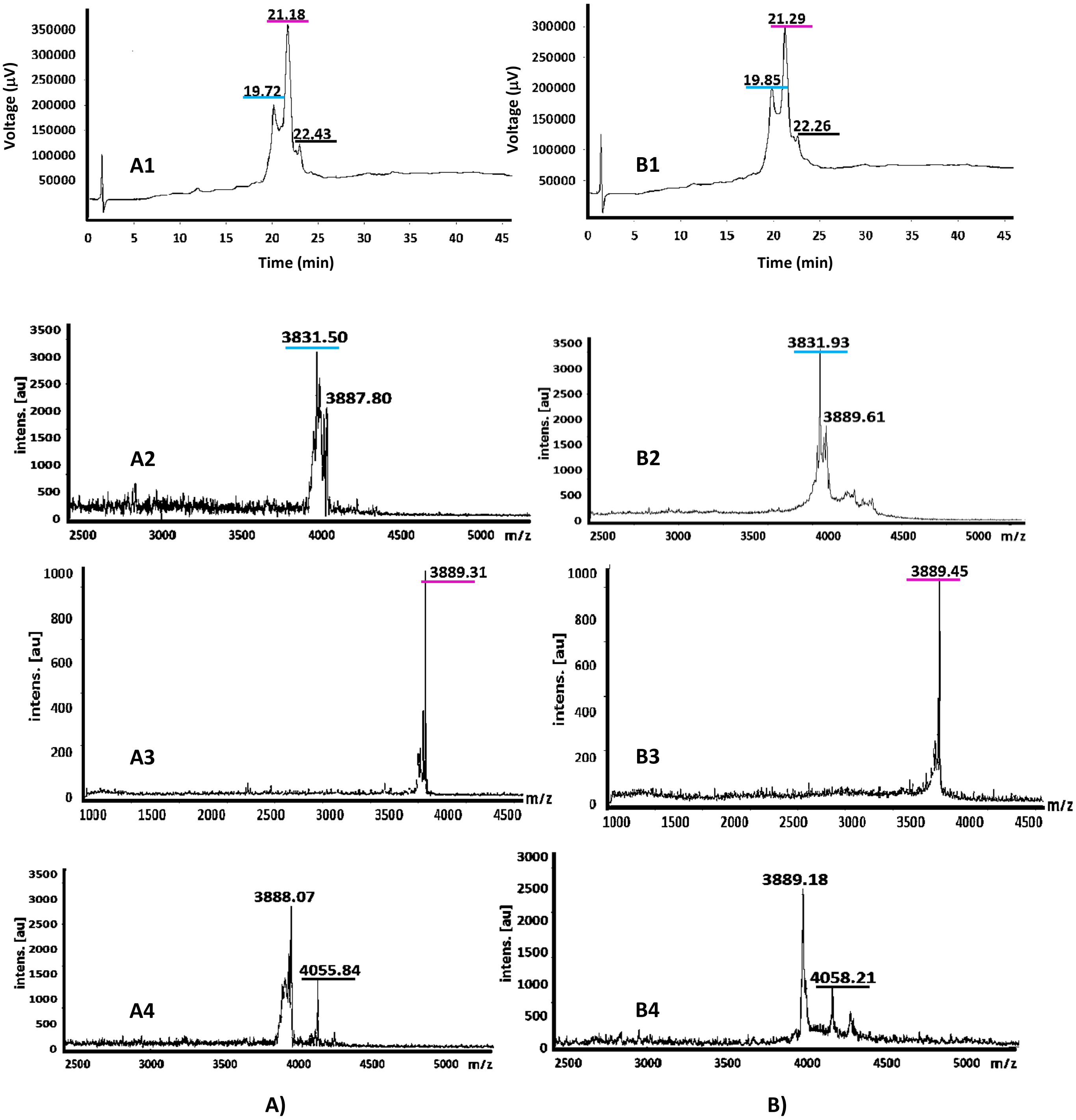

Figure S3. Chromatographic profiles of crude peptide's 33rd residue (3,887.86 Da theoretical) from standard (panel A1) and microwave-assisted reaction conditions (panel B1) and mass spectra for fractions for every peak in the chromatogram (A2 and B2, blue labelled t_R; A3 and B3, violet labelled t_R; and A4 and B4, black labelled)

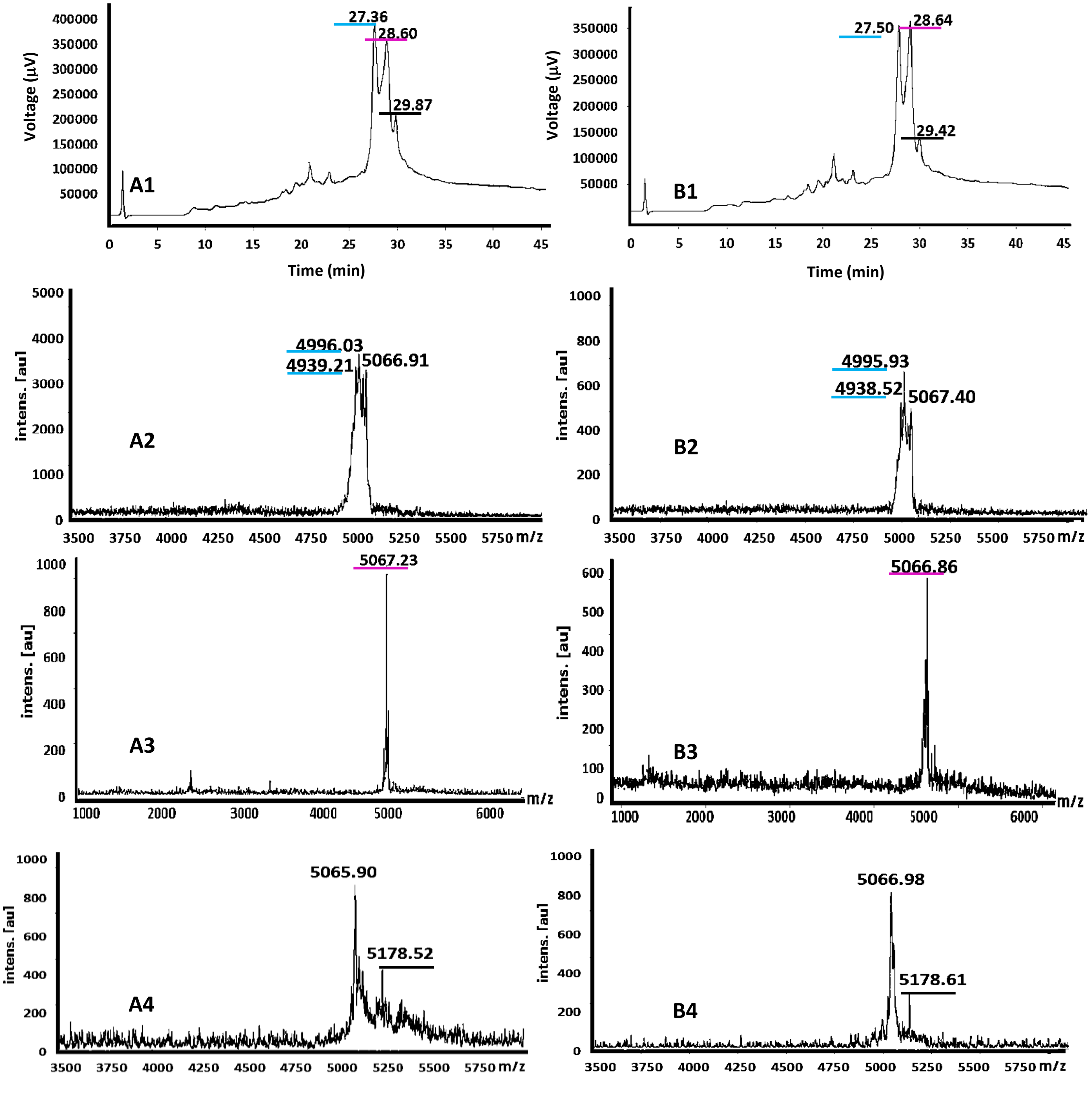

A) B)

Figure S4. Chromatographic profiles of crude peptide's 44th residue (5,065.51 Da theoretical) from standard (panel A1) and microwave-assisted reaction conditions (panel B1) and mass spectra of fractions for every peak in the chromatogram (A2 and B2, blue labelled t_R; A3 and B3, violet labelled t_R; and A4 and B4, black labelled)

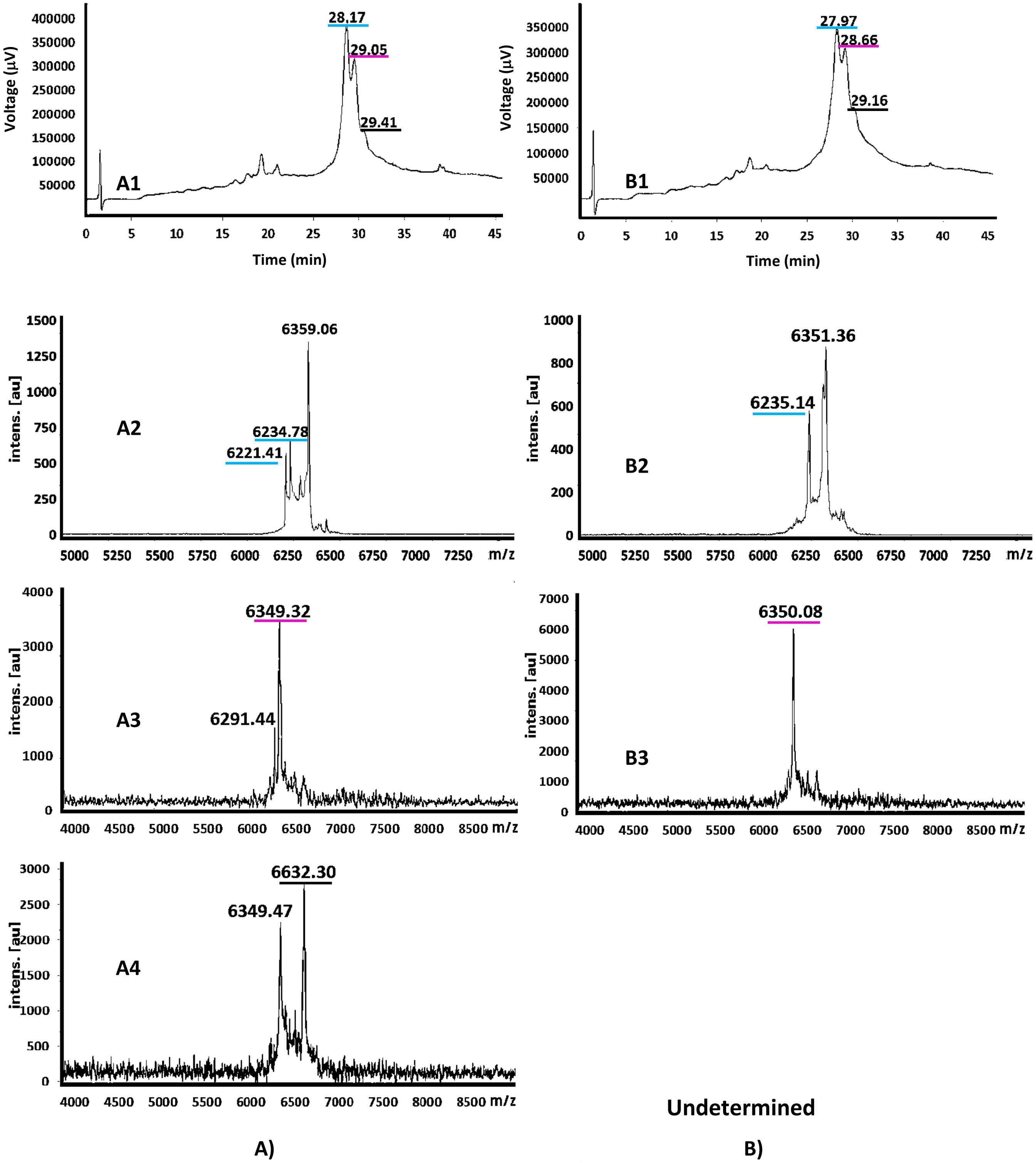

Figure S5. Chromatographic profiles of crude peptide's 57th residue (6,348.07 Da theoretical) from standard (panel A1) and microwave-assisted reaction conditions (panel B1) and mass spectra of fractions for every peak in the chromatogram (A2 and B2, blue labelled t_R; A3 and B3, violet labelled t_R; and A4, black labelled t_R)

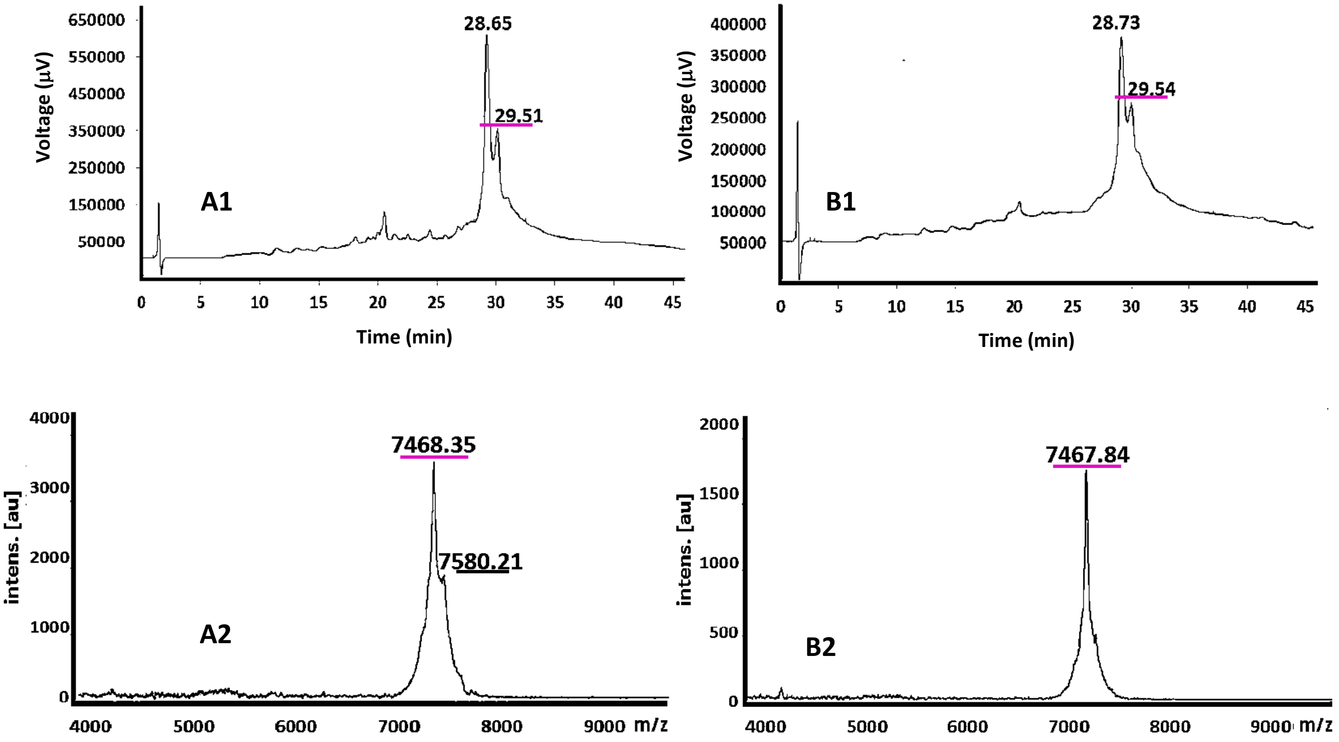

A) B)

Figure S6. Chromatographic profiles of crude peptide's 66th residue (7,467.68 Da theoretical) from standard (panel A1) and microwave-assisted reaction conditions (panel B1) and mass spectra of fractions for every peak violet labelled in the chromatogram (A2 and B2, labelled t_R)

Table S1. Molecular weight calculated for chimeric peptide and intermediary peptides

Residue number	Mono-isotopic molecular weight (Da)	Sequence
11	1,228.53	NYEEQPHISGG
22	2,628.16	MTDVNRYRYSNNYEEQPHISGG
23	2,685.18	GMTDVNRYRYSNNYEEQPHISGG
24	2,742.20	GGMTDVNRYRYSNNYEEQPHISGG
25	2,871.25	EGGMTDVNRYRYSNNYEEQPHISGG
26	2,984.33	LEGGMTDVNRYRYSNNYEEQPHISGG
30	3,481.66	LKKQLEGGMTDVNRYRYSNNYEEQPHISGG
33	3,887.86	YRSLKKQLEGGMTDVNRYRYSNNYEEQPHISGG
35	4,043.95	GVYRSLKKQLEGGMTDVNRYRYSNNYEEQPHISGG
36	4,114.99	AGVYRSLKKQLEGGMTDVNRYRYSNNYEEQPHISGG
39	4,424.19	VPLAGVYRSLKKQLEGGMTDVNRYRYSNNYEEQPHISGG
44	5,065.51	EVLYHVPLAGVYRSLKKQLEGGMTDVNRYRYSNNYEEQPHISGG
50	5,513.68	GGGYGGEVLYHVPLAGVYRSLKKQLEGGMTDVNRYRYSNNYEEQPHISGG
57	6,348.07	DYQAPFLGGGYGGEVLYHVPLAGVYRSLKKQLEGGMTDVNRYRYSNNYEEQPHISGG
60	6,703.24	FHADYQAPFLGGGYGGEVLYHVPLAGVYRSLKKQLEGGMTDVNRYRYSNNYEEQPHISGG
64	7,255.53	KHMRFHADYQAPFLGGGYGGEVLYHVPLAGVYRSLKKQLEGGMTDVNRYRYSNNYEEQPHISGG
66	7,467.68	VIKHMRFHADYQAPFLGGGYGGEVLYHVPLAGVYRSLKKQLEGGMTDVNRYRYSNNYEEQPHISGG
77	8,488.15	LGENPNANPGGVIKHMRFHADYQAPFLGGGYGGEVLYHVPLAGVYRSLKKQLEGGMTDVNRYRYSNNYEEQPHISGG
82	9,051.42	KNSFSLGENPNANPGGVIKHMRFHADYQAPFLGGGYGGEVLYHVPLAGVYRSLKKQLEGGMTDVNRYRYSNNYEEQPHISGG

The online *Peptide mass calculator* (http://www.ionsource.com/programs/PEPCALC.HTM) was used for calculating the above molecular weights

Table S2*:* Molecular weights calculated for amino acid and tert-butylated residues

Three- and one-letter code		Average molecular weight (Da)	Three- and one-letter code		Average molecular weight (Da)
Gly	G	57.05	Asp	D	115.10
Ala	A	71.08	Gln	Q	128.10
Ser	S	87.08	Lys	K	128.20
Pro	P	97.12	Glu	E	129.10
Val	V	99.07	Met	M	131.20
Thr	T	101.10	His	H	137.10
Cys	C	103.10	Phe	F	147.20
Leu	L	113.20	Arg	R	156.20
Ile	I	113.20	Tyr	Y	163.20
Asn	N	114.10	Trp	W	186.20

Number of tert-butylated residues	Average molecular weight (Da)
1	56.10
2	112.20
3	168.30
4	224.40
5	280.50
6	336.60
